# Forecasting *Brassica napus* production under climate change with a mechanistic species distribution model

**DOI:** 10.1038/s41598-023-38910-3

**Published:** 2023-08-04

**Authors:** Cláudia Eduarda Borges, Ronnie Von dos Santos Veloso, Crislaine Alves da Conceição, Débora Sampaio Mendes, Nadiezhda YZ Ramirez-Cabral, Farzin Shabani, Mahyat Shafapourtehrany, Marcela Carlota Nery, Ricardo Siqueira da Silva

**Affiliations:** 1 Universidade Federal dos Vales Jequitinhonha e Mucuri, Campus JK, Rodovia MGT 367 - Km 583, nº 5.000, Alto da Jacuba, Diamantina, MG CEP 39100-000 Brazil; 2 Ecosystem Management, School of Environmental and Rural Science, University of New England, Armidale, NSW 2351 Australia; 3 INIFAP, Campo Experimental Zacatecas, Km, 24.5 Carretera Zacatecas-Fresnillo, 98500 Calera de V.R., ZAC Mexico; 4 Department of Biological and Environmental Sciences, College of Arts and Sciences, Qatar University, P.O. Box 2713, Doha, Qatar; 5 Kandilli Observatory and Earthquake Research Institute, Department of Geodesy, Bogazici University, 34680 Cengelkoy, Istanbul, Turkey

**Keywords:** Biological models, Environmental sciences

## Abstract

*Brassica napus*, a versatile crop with significant socioeconomic importance, serves as a valuable source of nutrition for humans and animals while also being utilized in biodiesel production. The expansion potential of *B. napus* is profoundly influenced by climatic variations, yet there remains a scarcity of studies investigating the correlation between climatic factors and its distribution. This research employs CLIMEX to identify the current and future ecological niches of *B. napus* under the RCP 8.5 emission scenario, utilizing the Access 1.0 and CNRM-CM5 models for the time frame of 2040–2059. Additionally, a sensitivity analysis of parameters was conducted to determine the primary climatic factors affecting *B. napus* distribution and model responsiveness. The simulated outcomes demonstrate a satisfactory alignment with the known current distribution of *B. napus*, with 98% of occurrence records classified as having medium to high climatic suitability. However, the species displays high sensitivity to thermal parameters, thereby suggesting that temperature increases could trigger shifts in suitable and unsuitable areas for *B. napus*, impacting regions such as Canada, China, Brazil, and the United States.

## Introduction

*Brassica napus* L., commonly known as rapeseed or canola (short for Canadian oil low acid), is an oilseed crop belonging to the Brassicaceae family. It holds significant socioeconomic importance due to the oil’s high nutritional value, making it a valuable resource for human and animal nutrition^1–3^. This, in turn, generates substantial economic returns for producing countries^4^. In Canada, *B. napus* has emerged as one of the most extensively cultivated crops, contributing an impressive US$ 29.9 billion annually to the country's economy through its high oil production. The United States has also experienced a notable increase in *B. napus* oil production, with quantities rising from 486,251 tons in 2010 to 821,002 tons in 2020^5,6^. Consequently, the country witnessed a rise in imports to meet the surging demand for rapeseed oil, reaching 1827 million tons in 2020 compared to 1066 million tons in 2010^6^.

In the 2020/2021 period, global production of *Brassica napus* reached a substantial volume of 73.6 million tons (Mt)^7^. Projections indicate that the global supply is expected to witness a remarkable increase of 100.5 Mt in 2022/23^7^. When considering vegetable oil production, *B. napus* is the second-highest yielding oil crop worldwide, closely trailing soybeans. Recent harvests have yielded a significant quantity of 20–30 million metric tons of *B. napus* vegetable oil. Various countries, including members of the European Union, Canada, China, and India, have achieved notable oil production by cultivating this species. Notably, the European Union stands as the world's largest producer of *B. napus*, with a production volume of 19.5 million tons projected for the 2022/2023 harvest^1,4,7–9^. China and the European Union, recognized as important rapeseed producers, are expected to achieve annual outputs of 31 Mt and 30 Mt, respectively, by 2030^10^. Canada plays a significant role in rapeseed production, serving as the largest exporter of the crop and projecting an increase in production to 20 Mt by 2022/2023 and 23 Mt by 2030^7,10^. However, expanding the cultivation of *B. napus* presents challenges due to the lack of information about its cultivation and production in other regions. The species is highly susceptible to climatic variations. Simulations have revealed that the uncertainty surrounding the impact of climate change on rapeseed production intensifies over time, with the potential for significant increases in *B. napus* output in certain regions of China^11^.

Compared to other oil-producing crops like soybeans, there is still a scarcity of information regarding the optimal climatic conditions for cultivating *Brassica napus*. Soybean cultivation has well-established data for various regions across the globe. Consequently, there is a need to conduct further studies on *B. napus* to elucidate the specific climatic factors that contribute to its successful cultivation. Parameters such as luminosity, CO_2_ levels, rainfall, and temperature have been the focus of research to gain a better understanding of the cultivation requirements for this species^12–14^. These studies aim to fill the knowledge gaps and provide valuable insights into the climatic preferences and adaptability of *B. napus*, aiding farmers and researchers in optimizing its cultivation practices.

To address the uncertainties surrounding the ecological requirements and ecophysiological characteristics necessary for successful *Brassica napus* cultivation, the utilization of environmental niche models has proven to be a valuable approach^15–18^. These models offer a means to identify current and future areas that are suitable for cultivating *B. napus*, utilizing biological data obtained through field research^16,19–24^. By employing these models, it becomes possible to project areas with climates conducive to *B. napus* cultivation under various climate change scenarios^21^. Environmental niche models serve as a powerful tool in understanding the potential distribution and expansion of *B. napus*, assisting in decision-making processes related to agricultural practices, land management, and policy development. By incorporating a range of variables, such as temperature, precipitation, soil composition, and topography, these models provide insights into the ecological niche preferences of *B. napus* and offer predictions regarding its future distribution. By employing environmental niche models, researchers and policymakers can gain valuable information to guide crop management strategies and anticipate the impacts of climate change on *B. napus* cultivation. This approach contributes to the development of more informed and sustainable practices in the face of changing environmental conditions.

Climate change has introduced significant uncertainties regarding the future cultivation of various crop species, with potential adverse effects on their viability. This holds true for important crops such as *Zea mays* L., *Sorghum bicolor* L., and *Glycine max* L., as changes in temperature and precipitation patterns can pose significant challenges to their production^25–27^. As per Pullens et al.^14^, climate change is expected to bring about favorable conditions for winter *Brassica napus* cultivation in Europe's northern regions, specifically within the boreal environmental zone, due to rising temperatures. Consequently, modeling techniques have become indispensable tools for studying *B. napus*. These models enable the simultaneous assessment of species distribution across diverse locations, encompassing both spatial and temporal dimensions. They play a crucial role in understanding the potential effects of climate change on *B. napus* distribution and cultivation. To enhance our understanding of *B. napus* production under changing climatic conditions, it is crucial to focus on research that investigates the specific impacts of climate change on this crop. Such studies can provide valuable insights into the areas and mechanisms through which *B. napus* cultivation is likely to be most affected by climate change^14^. By examining how and where the crop could be susceptible to climatic shifts, we can better prepare for potential challenges and develop adaptive strategies to mitigate the impacts on *B. napus* production. Overall, employing modeling approaches and conducting targeted research on the effects of climate change are vital steps toward enhancing our understanding of *B. napus* cultivation in the face of evolving environmental conditions. These efforts contribute to the development of effective adaptation and mitigation strategies to ensure the resilience and sustainability of *B. napus* production systems.

The prediction of species distribution can be achieved through the utilization of correlative and mechanistic predictive models^18,28^. Correlative models rely on environmental predictor variables that are closely associated with species distribution records, allowing for predictions to be made based on these associations^18,29^. On the other hand, mechanistic models go beyond species distribution records and incorporate more detailed information on the biophysical and physiological responses of the species. These models typically draw upon specific research findings to define the environmental predictor variables^18,28,30^. Given the objectives of the study and the existing knowledge about *Brassica napus*, a mechanistic model, specifically CLIMEX, was employed in this research. Mechanistic models offer the advantage of incorporating detailed biophysical and physiological information, which is valuable for understanding the species’ responses to environmental factors^17,30–35^. CLIMEX, in particular, has demonstrated good performance and is increasingly being applied in various studies. By utilizing a mechanistic model like CLIMEX, this study can provide more nuanced insights into the potential distribution of *B. napus*. The incorporation of biophysical and physiological responses enhances the accuracy and reliability of the predictions, allowing for a more comprehensive understanding of the species’ ecological niche and its response to changing climatic conditions.

The study of climate change’s impact on *Brassica napus* is of paramount importance due to its wide-ranging implications for agricultural productivity, food security, biodiversity, and carbon sequestration. Changes in temperature and precipitation patterns can significantly influence the growth, development, and yield of *B. napus*, making it essential to understand how climate change affects this crop. Such understanding enables farmers and policymakers to proactively prepare for potential shifts in agricultural productivity, ensuring sustainable food production systems^36–39^. *Brassica napus* plays a crucial role in global food security as a vital source of vegetable oil and protein for human consumption and animal feed. Any disruption in its production, caused by climate change, can have severe implications, particularly in developing countries heavily reliant on this crop. Therefore, studying the effects of climate change on *B. napus* is vital for safeguarding food security at both regional and global levels^37,40^. Furthermore, *B. napus* is an integral component of natural ecosystems, providing food and habitat for various organisms. Climate change can alter the distribution and abundance of this species, potentially impacting biodiversity. Understanding the species’ responses to changing climatic conditions aids in predicting and mitigating the cascading effects on other organisms that depend on *B. napus* for their survival and ecological interactions^41^. Additionally, *B. napus* exhibits a remarkable capacity for carbon sequestration. As a fast-growing crop, it can absorb substantial amounts of atmospheric carbon dioxide (CO_2_) and store it in its biomass and soil. Examining the effects of climate change on *B. napus* is instrumental in assessing its potential as a tool for mitigating greenhouse gas emissions and promoting carbon sequestration strategies^4,42^. Given the current lack of comprehensive understanding regarding the changing climatic factors associated with *B. napus*, both in the present and the future, this study aims to develop a potential ecological niche distribution model. By utilizing modeling tools, it seeks to evaluate the species' sensitivity to climatic factors and provide valuable insights into its ecological requirements under changing environmental conditions. This research will contribute to bridging the knowledge gap and enable informed decision-making in the context of climate change and *B. napus* cultivation^13,43,44^.

## Material and methods

### Brassica napus distribution

**Figure 1:**
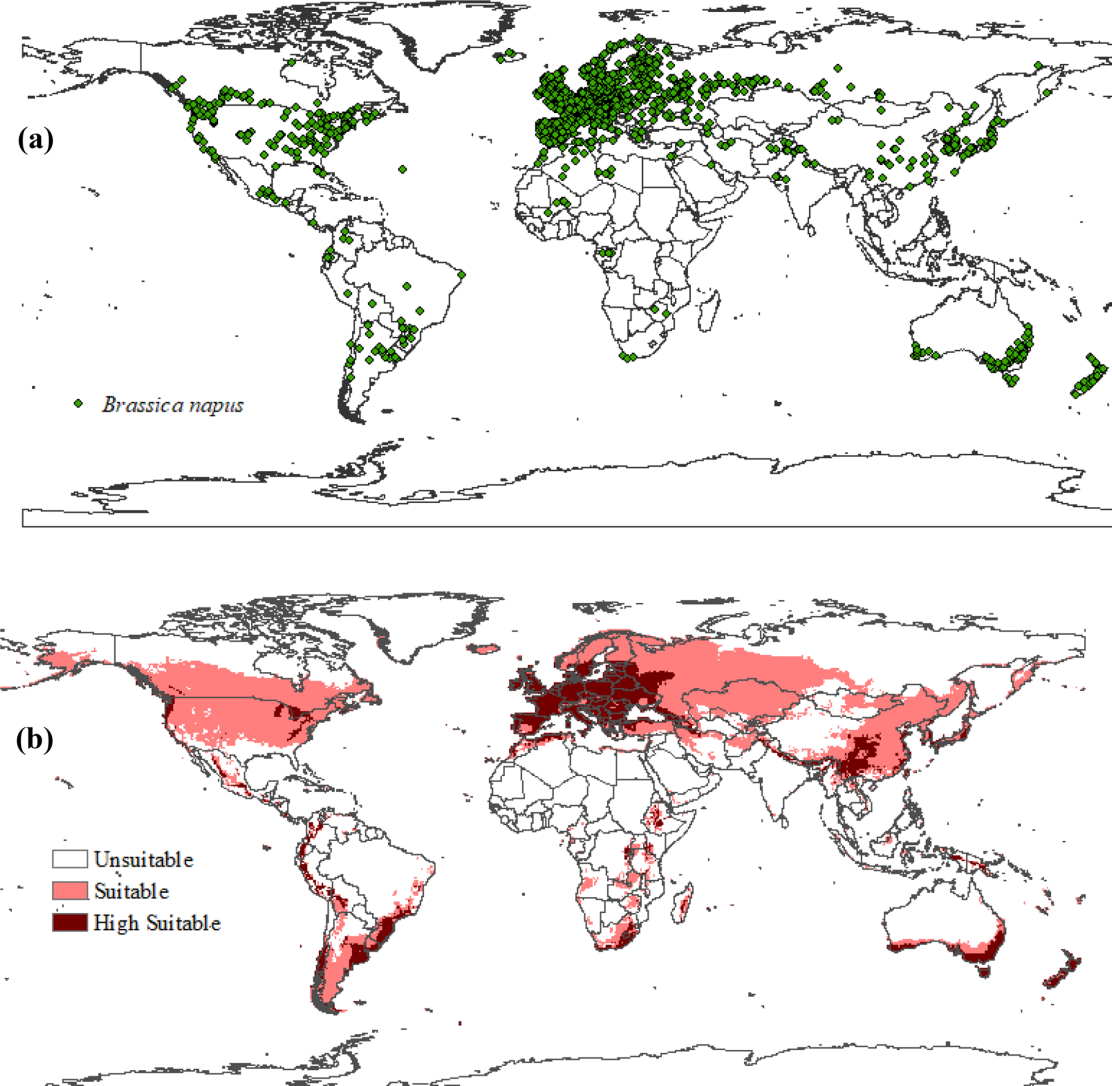
(**a**) *Brassica napus* occurrence worldwide. (**b**) Ecoclimatic index (EI) for the current climate scenario of *B. napus* modeled using CLIMEX. The areas unsuitable in white (EI = 0), suitable in pink (0 < EI < 30) and high suitable in dark red (30 < EI < 100).

The geographic occurrence of *Brassica napus* L. was comprehensively characterized through a systematic survey conducted across multiple data sources. The survey encompassed the Global Biodiversity Information Facility (GBIF) website (http://www.gbif.org)^45^, the European and Mediterranean Plant Protection Organization (EPPO), and the Center for Agriculture and Biosciences International (CABI), utilizing the keyword “*Brassica napus*.” Additionally, a thorough literature search was conducted using Google Scholar, incorporating keywords such as “*Brassica napus*,” “canola,” or “rapeseed,” representing the scientific and common names of the species (Supplementary Information). The search was conducted in May 2022, resulting in the identification of 62,797 records. To ensure data quality and relevance, a series of data cleaning steps were performed. Records without coordinates were removed, resulting in the elimination of 29,057 records. Duplicate records were also identified and excluded, resulting in the removal of 9542 records. Additionally, records located within a 50 km radius were considered equidistant and reduced to a single record, resulting in the removal of 22,611 records. Ultimately, a total of 1587 records of *B. napus* were utilized for constructing the species distribution model. For the purpose of model validation, a subset of 1515 records (64% of the 1587 records) was randomly selected, ensuring a representative sample. These records were used to evaluate the performance and accuracy of the model (Fig. 1). By employing rigorous data collection and validation procedures, this study ensures robust and reliable input for the species distribution model, facilitating accurate assessments of *B. napus*’ geographic occurrence and providing a solid foundation for subsequent analyses and predictions.

### CLIMEX description

The models utilized in this study were implemented using CLIMEX version 4.0 software^46^. CLIMEX offers a powerful framework for comparing locations and time periods, enabling the creation of maps that illustrate changes in the climatic suitability of various species over space and time. By incorporating climatic parameters derived from species-specific biological information and geographic distribution data, CLIMEX facilitates the simulation and estimation of potential areas conducive to the growth and development of target species. One of the key strengths of CLIMEX is its ability to generate insights into the spatiotemporal dynamics of species under current climatic conditions as well as future scenarios considering climate change. By leveraging its functionality, researchers can investigate the distribution patterns and climatic preferences of species in a comprehensive and detailed manner. Through the utilization of CLIMEX, this study has been able to simulate and assess the potential ecological niche distribution of *Brassica napus* L. under current conditions and future scenarios influenced by climate change. The software’s analytical capabilities enable the exploration of suitable areas for the species and facilitate the study of how its distribution may shift over time in response to changing climatic conditions. By leveraging CLIMEX's robust modeling capabilities, this research contributes to enhancing our understanding of the spatiotemporal dynamics and ecological responses of *Brassica napus L.* to climate change, thereby providing valuable insights for effective species management and conservation in the face of ongoing environmental challenges.

### CLIMEX parameter adjustment

The development of the CLIMEX model for *Brassica napus* involved the utilization of key parameters derived from biological data that characterize the species’ growth requirements under both favorable and unfavorable conditions. These parameters primarily focused on temperature and humidity, encompassing maximum and minimum thresholds conducive to the optimal growth of *B. napus*. Conversely, indices related to stress factors such as cold, heat, dryness, and humidity were also considered to determine ranges where the species would not thrive or develop adequately. To ensure accuracy and relevance, the selection of these parameters was based on a thorough review of recent literature pertaining to the biological characteristics of *B. napus*. By incorporating up-to-date information, the model's parameters were fine-tuned to reflect the species' specific needs and constraints. In total, sixteen parameters were established, encompassing various aspects of *B. napus*’ ecological requirements. These parameters formed the foundation for identifying and delineating the potential distribution of ecological niches suitable for *B. napus* across different geographic regions. In order to refine and validate the model, the initially defined parameters were further adjusted based on the observed current distribution of *B. napus*. By comparing the model’s predictions with the actual occurrence of the species, necessary refinements and calibrations were made to enhance the model's accuracy and reliability. By employing this rigorous approach in parameter selection and adjustment, the CLIMEX model for *B. napus* offers a robust framework for predicting the potential distribution of ecological niches and understanding the species' response to varying environmental conditions.

#### Temperature index

The temperature indices utilized in the model play a crucial role in establishing the suitable temperature range for the growth of *Brassica napus*. These indices include DV0 (lower temperature limit), DV1 (lower optimum temperature), DV2 (upper optimum temperature), and DV3 (upper-temperature limit) as described by Morrison et al.^47^. DV0 represents the minimum temperature required for the plant's growth. According to Morrison et al.^47^, the base temperature for *Brassica napus* is determined to be 5 °C. Below this threshold, the species does not achieve the necessary developmental percentage for physiological maturity. For optimal development, *B. napus* thrives around 20 °C, with a temperature range of 12–30 °C^48^. However, higher temperatures above 27 °C can lead to plant sterility and floral abortion, as reported by Morrison^49^ and other studies^50,51^. Consequently, DV3, representing the maximum temperature for plant growth, was set at 27 °C. DV1 and DV2 denote the lower and upper ideal temperatures, respectively, for the species' development. After fine-tuning the model using occurrence data from North America, these temperatures were determined to be 10 °C and 23 °C, respectively, better aligning with the species’ distribution in that region (Table 1). By incorporating these temperature indices and their specific values into the model, the CLIMEX model can accurately capture the temperature requirements for optimal growth and development of *Brassica napus*, facilitating predictions and analyses of its potential ecological niche under varying temperature conditions.

**Table 1:** CLIMEX parameter values used for modeling the distribution of *Brassica napus* (value model) and CLIMEX Parameters values included in the sensitivity analysis of *B. napus* (Low and high value) according to the methodology of Kriticos et al.^46^.

Parameter	Acronym	Low value	Value model	^(^*^)^ References	High value
Lower temperature limit (°C)	DV0	4	5	^1^	6
Lower optimal temperature (°C)	DV1	9	10	^2,3^	11
Upper optimal temperature (°C)	DV2	22	23	^3^	24
Upper temperature limit (°C)	DV3	26	27	^1^	28
Limiting low soil moisture	SM0	0.09	0.1	**	0.11
Lower optimal soil moisture	SM1	0.18	0.2	**	0.22
Upper optimal soil moisture	SM2	0.72	0.8	**	0.88
Limiting high soil moisture	SM3	2.25	2.5	^4^	2.75
Cold stress temperature threshold (°C)	TTCS	0.9	1	**	1.1
Cold stress temperature rate (week^−1^)	THCS	− 0.000081	− 0.00009	**	− 0.000099
Heat stress temperature threshold (°C)	TTHS	26	27	^1^	28
Heat stress accumulation rate (week^−1^)	THHS	0.0009	0.001	**	0.0011
Dry stress threshold	SMDS	0.09	0.1	**	0.11
Dry stress rate (week^−1^)	HDS	− 0.0045	− 0.005	^4^	− 0.0055
Wet stress threshold	SMWS	2.25	2.5	^4^	2.75
Wet stress rate (week^−1^)	HWS	0.0018	0.002	^4^	0.0022

Moisture value units are dimensionless indices in proportions of soil moisture-holding capacity. *Reference value model: ^1^Morrison et al.^49^, ^2^Kriticos et al.^46^, ** Values adjusted to species distribution.

#### Cold and heat stress

*Brassica napus* is inherently a temperate to hard-temperate crop^48^. In order to account for the species’ ability to withstand adverse conditions, stress indices are incorporated in the CLIMEX model. These indices aim to limit the species’ survival capacity during unfavorable circumstances. The cold stress temperature threshold (TTCS) represents the average of the minimum weekly temperatures that the species can tolerate, while the cold stress temperature rate (THCS) signifies the accumulation of cold stress when the minimum temperature falls below the value of TTCS^46^. Based on the research conducted by Thomas^48^, it was determined that the base temperature for *Brassica napus* ranges from 0 to 5 °C. Consequently, TTCS and THCS were set at 1 °C and − 0.00009, respectively, to better align with the occurrence data of *B. napus* (Table 1). Heat stress in the model is determined by the heat stress temperature threshold (TTHS) and the heat stress temperature rate (THHS). TTHS represents the temperature threshold at which heat stress begins to impact the species, while THHS quantifies the rate at which stress accumulates when temperatures exceed the TTHS value^46^. High temperatures have a significant influence on the flowering time and pollen viability of *B. napus*, as observed by Morrison^49^ at temperatures above 27 °C. Therefore, TTHS and THHS were established at 27 °C and 0.001, respectively. The selection of THHS was based on the recommended value for temperate crops in the CLIMEX manual and its compatibility with the occurrence data of *B. napus* in North America (Table 1). By incorporating these stress indices into the CLIMEX model, it becomes possible to capture the species' response to cold and heat stress, providing a comprehensive understanding of its ecological niche and enhancing the accuracy of predictions related to its distribution and viability under various climatic conditions.

#### Moisture index

Soil moisture parameters play a crucial role in the CLIMEX model, governing the population growth of the species in response to humidity. The four parameters used to define soil moisture in CLIMEX are Limiting low soil moisture (SM0), Lower optimal soil moisture (SM1), Upper optimal soil moisture (SM2), and Limiting high soil moisture (SM3). These parameters are essential for understanding the species' population dynamics under varying moisture conditions. Within the range of SM1 and SM2, the population growth of the species is maximized, indicating the ideal soil moisture levels for *Brassica napus*. When the soil moisture falls below SM0 or exceeds SM3, population growth becomes null, suggesting the thresholds beyond which the species cannot thrive^46^. *Brassica napus* has relatively low water requirements throughout its life cycle, typically necessitating an average annual rainfall of above 450 mm^52^. Excessive water can negatively impact grain yield and oil content in *B. napus*^48^. While water demand is generally low, water deficit during flowering stages can adversely affect grain yield and oil content^53^. Moreover, an excess of water can lead to a reduction in the number of silique branches developed per plant^54^. To model the soil moisture aspect, the moisture indices SM0, SM1, SM2, and SM3 were determined as 0.1, 0.2, 0.8, and 2.5, respectively. These values were chosen based on the recommended guidelines for temperate crops provided in the CLIMEX user’s guide^46^. Additionally, they were fine-tuned to align with the occurrence data of *B. napus* in North America, ensuring a more accurate representation of the species' ecological niche and responses to different soil moisture levels (Table 1).

#### Dry and wet stress

Soil moisture plays a critical role in the well-being of plants, and extreme moisture conditions, either too dry or too wet, can cause stress to the plant. In the CLIMEX model, this stress is quantified using parameters such as the dry stress threshold (SMDS) and the wet stress threshold (SMWS), along with the dry stress rate (HDS) and wet stress rate (HWS)^46^. For *Brassica napus*, the flowering period is particularly susceptible to dry stress, which can significantly reduce the crop's oil yield. Conversely, high soil humidity can also lead to yield losses of up to 50% when compared to well-drained soils^48^. To capture these effects in the model, the stress indices SMDS, SMWS, HDS, and HWS were set at specific values. The SMDS and SMWS were defined as 0.1 and 2.5, respectively, based on the corresponding values of SM0 and SM3 used for *B. napus*. These thresholds indicate the soil moisture levels below which dry stress or above which wet stress occurs. The HDS and HWS, representing the rates at which stress accumulates in response to dry or wet conditions, were determined as − 0.005 and 0.002, respectively. These parameter values were selected based on the recommendation provided in the CLIMEX manual for temperate crops and through careful fitting to the occurrence data of *B. napus* in North America (Table 1). By incorporating these moisture stress indices, the model can more accurately simulate the species’ responses to different soil moisture conditions, providing valuable insights into the potential ecological niches of *B. napus*.

### Ecoclimatic index, historical meteorological data, and model validation

The climate suitability model for *Brassica napus* was developed using the ecoclimatic index (EI), a comprehensive annual index that combines the species' growth index (favorable conditions) and stress index (unfavorable conditions). The EI ranges from 0 to 100, where values close to 0 indicate unsuitability for species growth, and an EI of 100 represents the ideal climatic conditions for growth. However, it’s important to note that an EI of 100 is typically found in controlled climatic environments^46^. In this study, the regions were categorized into three classes based on the EI values: inadequate regions (EI = 0), adequate regions (EI ranging from 0 to 30), and regions with high suitability (EI ≥ 30)^46^. This classification provides a useful framework for understanding the potential suitability of different regions for *B. napus* cultivation. These inferred parameters can then be utilized to project the potential range of the species in new areas or under different climate scenarios. To develop the model, climate data from the CliMond 10′ grid was employed. Historical adequacy was modeled based on maximum and minimum monthly average temperatures, monthly average precipitation, and relative humidity at 9:00 h and 15:00 h, covering the period from 1961 to 1990. This 30-year period centered on 1975 was chosen as a representative time frame for modeling the historical suitability of *B. napus*^46^. To validate the model, a reserved region with occurrence records of the species, which were not used for parameter adjustment, was utilized. After parameter fitting, the suitability of the reserved region was assessed to verify if it aligned with the known occurrences of the species. In this study, the European continent was selected as the validation region for the model. By employing the EI and incorporating historical climate data, this modeling approach provides insights into the suitability of different regions for *B. napus* cultivation, aiding in understanding the potential ecological niche distribution of the species.

### Climate change scenario

The The Intergovernmental Panel on Climate Change (IPCC) report, specifically the Fifth Assessment Report (AR5), introduces four representative concentration pathways (RCPs) as greenhouse gas trajectories to replace the SRES (Special Report on Emissions Scenarios) scenarios^55^. These RCPs, namely RCP 2.6, RCP 4.5, RCP 6.0, and RCP 8.5, represent different levels of energy absorption by the Earth's atmosphere and the subsequent energy reflection back into space, measured in watts per square meter. RCP 8.5 is a high-emission scenario where radiative forcing is projected to reach 8.50 W/m^2^ by 2100^56^. This scenario assumes factors such as significant population growth and sustained lower incomes in developing countries. To assess the future suitability of *Brassica napus* under climate change, the RCP 8.5 emission scenario was utilized. The study focused on the period from 2040 to 2059, centered on 2050, using two climate models: Access 1.0 and CNRM-CM5. Climate data with a resolution of 30 min (30′) were obtained from the CliMond database (www.climond.org). RCP 8.5 is a high-emission scenario that shares similarities with the SRES A1FI scenario, which projects high levels of greenhouse gas emissions^57^. By employing these climate models and data, the study aimed to evaluate the species' potential suitability under future climate conditions characterized by high greenhouse gas emissions. This information contributes to our understanding of how *Brassica napus* may respond and adapt to different climate scenarios, assisting in climate change impact assessments and informing mitigation and adaptation strategies.

### Parameter sensitivity in CLIMEX

A sensitivity analysis was conducted to assess the impact of each parameter on the model's output, revealing varying degrees of sensitivity among the parameters. The analysis involved adjusting each parameter to both low and high values according to the instructions provided by the CLIMEX software. Temperature parameters were adjusted by ± 1 °C, while soil moisture parameters, stress indices, and stress rates were adjusted by ± 10%^46^. In total, 16 parameters were used in this study. To perform the sensitivity analysis, the model was executed 32 times, each time with different parameter values. The ecoclimatic index (EI) was used to evaluate the changes in each region resulting from the adjustments to parameter values. The parameters that exhibited the most significant influence on the model's output were categorized as “sensitive,” while those with minimal impact were labeled as “insensitive.” The potential changes resulting from adjusting each parameter were assessed in different categories: inadequate, adequate, and high adequacy, to measure the sensitivity levels of the parameters in terms of area changes. By readjusting the parameters, the EI values in each adequacy category were either increased or decreased. Positive percentages indicate an increase in EI, while negative percentages indicate a reduction in EI. This thorough sensitivity analysis provides valuable insights into the relative importance of each parameter and its influence on the model's outcomes. By understanding the sensitivity levels, researchers can prioritize and focus on the parameters that have the most substantial impact, enhancing the reliability and robustness of the climate suitability model for *Brassica napus*.

## Results

### Geographic distribution of *B. napus* and model validation

The analysis of occurrence records revealed the distribution of *Brassica napus* across various countries (Fig. 1a). The majority of *B. napus* records occur in Europe, accounting for 64% of the occurrences (1015 records). In contrast, the African continent represents only 2% of the species' records (32 records). The American continent contributes 15% (238 records), with a concentration in the northern region. The Australian and Asian continents have 9% and 10% of *B. napus* records, respectively, amounting to 143 and 159 occurrences (Fig. 1a). The model successfully demonstrates a good correspondence between the actual distribution of *B. napus* and the potential distribution modeled for the species. Approximately 98% of the occurrence records are located in regions that exhibit climatic suitability for the species. Among these records, 72% are situated in regions with high suitability (EI ≥ 30), while 26% are in areas with moderately suitable conditions (0 < EI < 30). The validation process focused on the European continent due to the abundance of *B. napus* occurrence records in this area. Comparing the actual distribution (occurrence records) with the potential distribution (climate suitability) in the validation area, we find that 100% of the occurrence records in Europe align with regions exhibiting climatic suitability. This validation process confirms the effectiveness of the selected parameter values in the CLIMEX model (Fig. 1). Overall, the analysis demonstrates a strong agreement between the model's predictions and the observed distribution of *B. napus*, indicating the reliability and accuracy of the model in assessing climate suitability for the species.

### Regions with climatic suitability for *B. napus*

The modeling results reveal that a significant portion of the United States territory is suitable for cultivating *Brassica napus*, accounting for more than half of the country’s land area. Furthermore, several regions in Mexico, Guatemala, Costa Rica, Colombia, Venezuela, Ecuador, Peru, Bolivia, Chile, South and Southeast Brazil, the entirety of Argentina, Canada, the European continent, and Uruguay exhibit high suitability for *B. napus* cultivation (Fig. 1b). In Africa, countries such as South Africa, Zimbabwe, Angola, Zambia, Malawi, Tanzania, Kenya, Uganda, Rwanda, Burundi, Ethiopia, and Madagascar display suitable and highly suitable climatic conditions. Likewise, regions in Russia, China, Turkey, Georgia, Azerbaijan, Kazakhstan, Iran, Afghanistan, South and North Korea, Japan, South Australia (Coasts), and New Zealand offer suitable and highly suitable areas for cultivating *B. napus*.

Conversely, the Sahara Desert, central Australia, significant portions of Canada, over 80% of the Brazilian territory, Paraguay, Venezuela, and parts of Russia are among the countries or regions that do not present climatic suitability (EI = 0) for *B. napus* growth (Fig. 1b). These findings provide valuable insights into the potential geographic distribution of suitable climate conditions for *B. napus* cultivation, assisting policymakers, farmers, and researchers in identifying favorable areas for crop production and optimizing resource allocation for sustainable agricultural practices.

### Sensitivity analysis

**Figure 2:**
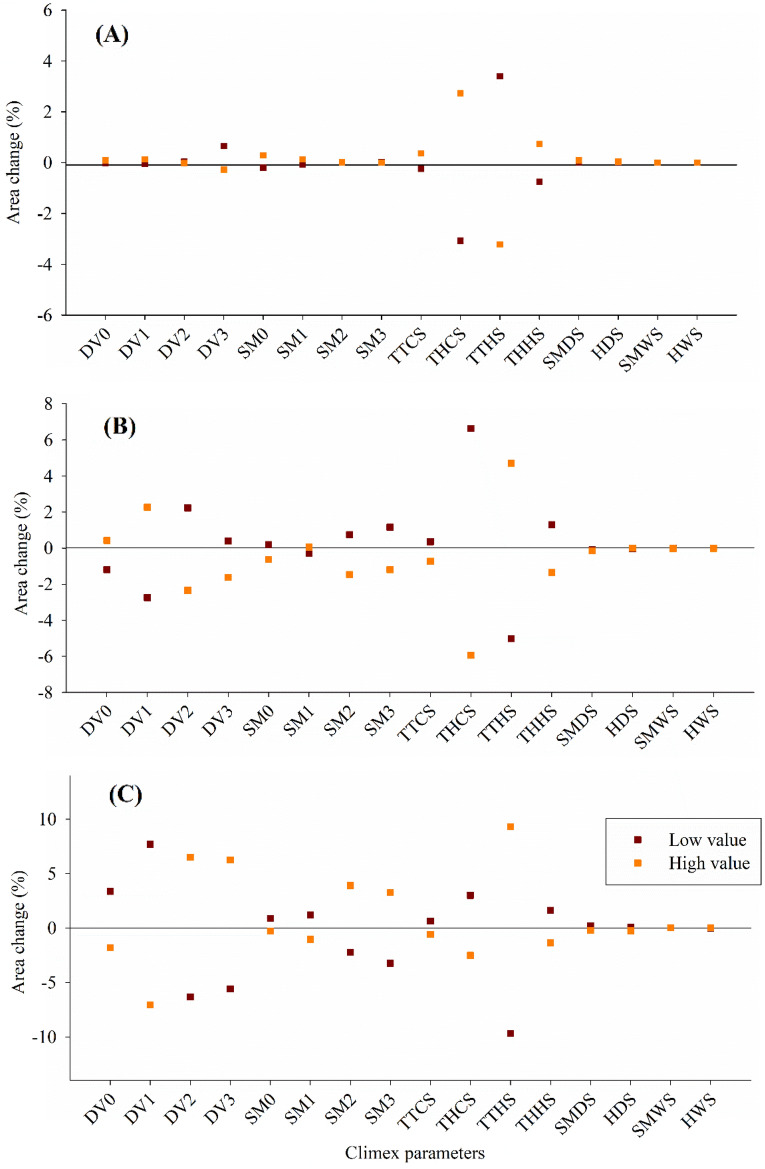
Area changes in (**A**) unsuitable, (**B**) suitable areas, and (**C**) high suitable areas for the potential distribution of *Brassica napus* when sensitivity analysis was undertaken based on 16 CLIMEX parameters with higher sensitivity in EI. The values for the parameters used are those given in Table 1. DV0—lower temperature limit, DV1—lower optimum temperature, DV2—upper optimum temperature, DV3—upper-temperature limit, SM0—Limiting low soil moisture, SM1—Lower optimal soil moisture, SM2—Upper optimal soil moisture, SM3—Limiting high soil moisture, TTCS—Cold stress temperature threshold, THCS—Cold stress temperature rate, TTHS—Heat stress temperature threshold, THHS—Heat stress accumulation rate, SMDS—Dry stress threshold, HDS—Dry stress rate, SMWS—Wet stress threshold, HWS—Wet stress rate.

The sensitivity analysis conducted on the model revealed that certain parameters had a greater impact than others. In regions with unsuitable climatic conditions (EI = 0), the most sensitive parameters were THCS, TTHS, and THHS. Increasing THCS to a high value (− 0.000099) and setting TTHS to a low value (26 °C) resulted in an approximately 6% increase in unsuitable areas. Similarly, when THHS was adjusted to a high value (0.0011), the unsuitable area increased by 4%. Generally, readjusting all parameters contributed to expanding the unsuitable climate areas (Fig. 2a). Significant reductions in suitable climatic regions (EI from 0 to 30) were observed when individual model parameters were altered. The most sensitive parameters that led to decreases in suitable climatic areas were DV1 (low value of 9 °C), DV2 (high value of 24 °C), THCS (high value of − 0.000099), and TTHS (low value of 26 °C), resulting in reductions of 9.3%, 8.9%, 12.3%, and 11.4%, respectively (Fig. 2b). Regions with high climatic suitability were found to be more sensitive to variations in model parameters. Adjusting all parameters resulted in significant increases and decreases in high suitability regions, depending on the specific parameter. Decreasing the value of parameters DV1 (low value of 9 °C), DV2 (high value of 24 °C), DV3 (high value of 28 °C), and TTHS (high value of 28 °C) increased the high suitability areas. Conversely, increasing the value of parameters DV1 (high value of 11 °C), DV2 (low value of 22 °C), DV3 (low value of 26 °C), and TTHS (low value of 26 °C) led to decreases in areas with high climatic suitability, resulting in reductions of 9.2%, 8.4%, 7.7%, and 11.7%, respectively (Fig. 2c). These sensitivity analyses provide valuable insights into the relative importance of different parameters in the model and their impact on unsuitable, suitable, and highly suitable climatic regions for *Brassica napus* cultivation. The findings highlight the need for careful consideration and accurate calibration of these parameters to ensure the reliability and robustness of the climate suitability model.

### *Brassica napus* climate suitability in climate change scenarios

**Figure 3:**
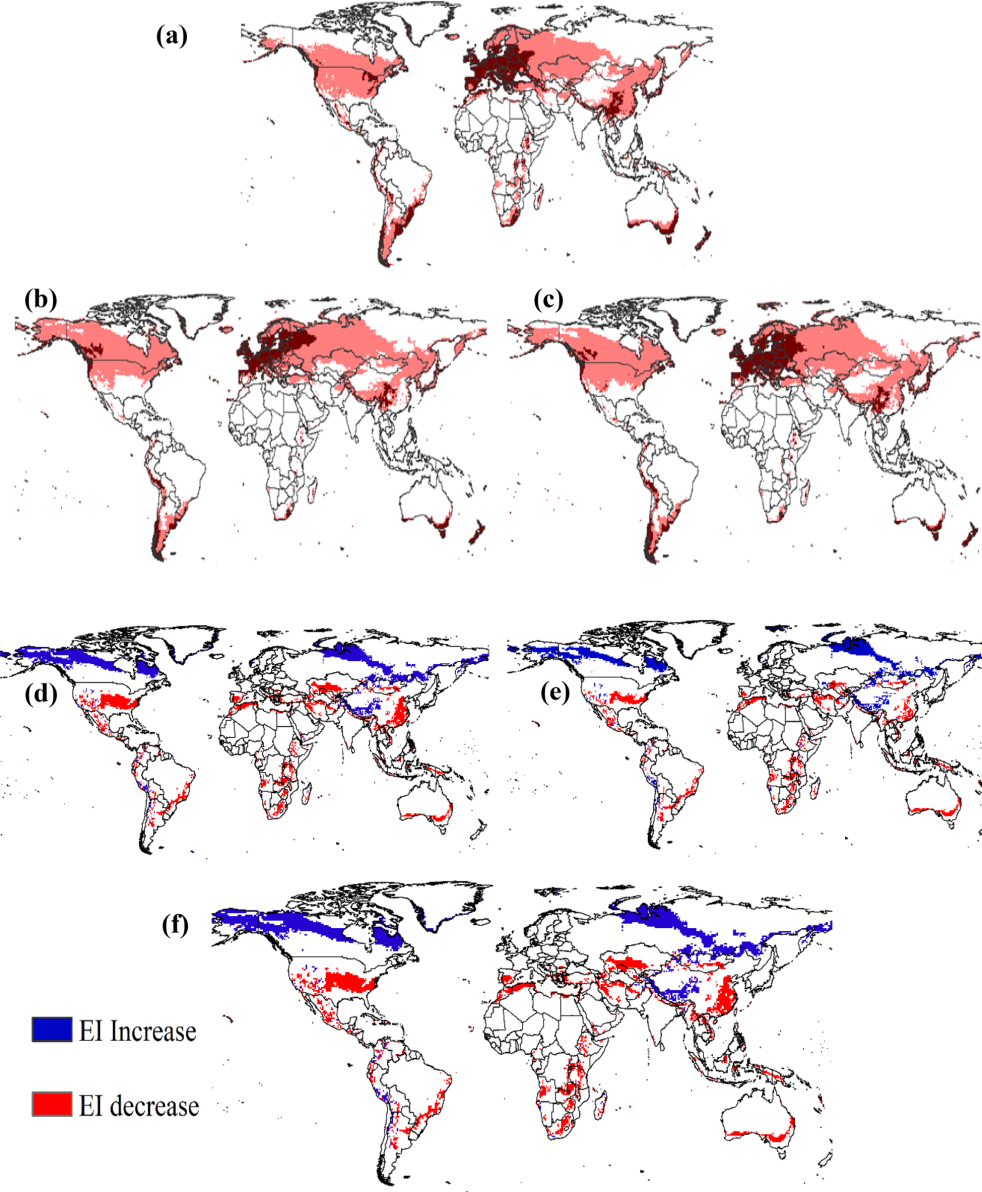
Ecoclimatic index (EI) for *Brassica napus* in different years (**a**) present time (**b**) 2040–2059 under Access 1.0 model, scenario RCP 8.5 (**c**) 2040–2059 under CNRM-CM5 model, scenario RCP 8.5 (**d**) agreement in EI alterations between Access 1.0 and present time (**e**) agreement in EI alterations between CNRM-CM5 model and present time (**f**) agreement in EI alterations between model access 1.0 model with CNRM-CM5 model.

The analysis of future scenarios with climate change (2040–2059) revealed both reductions and increases in areas with climate suitability for *B. napus* cultivation across various countries worldwide (Fig. 3). Comparing the two models, Access 1.0 (Fig. 3b,d) showed a more pronounced reduction in suitable areas in the United States compared to the CNRM-CM5 model under the RCP 8.5 Scenario (Fig. 3c,e). Conversely, Canada experienced an increase in suitable climatic conditions in the Access 1.0 projection (Fig. 3b,d) compared to the CNRM-CM5 model (Fig. 3c,e).

On the European continent, areas with high climatic suitability showed a decrease with climate change in the RCP 8.5 scenario in both the CNRM-CM5 model (Fig. 3c) and the Access 1.0 model (Fig. 3b). Notably, some European countries transitioned from high suitability to suitable conditions, with Access 1.0 (Fig. 3b) displaying a more significant change compared to CNRM-CM5 (Fig. 3c).

Figure 3f presents regions that may experience an increase or decrease in EI for *B. napus* with climate change in the two models (Access 1.0 vs. CNRM-CM5) under the RCP 8.5 emission scenario between 2040 and 2059. Southern Peru, parts of Canada and Russia, as well as areas in Mongolia and China, were projected to have an increase in EI, indicating improved climate suitability for *B. napus*. However, losses in climate suitability were projected across continents where *B. napus* is cultivated. In the Americas, reductions were anticipated in the United States, Mexico, Guatemala, Honduras, Colombia, Venezuela, Peru, Ecuador, Brazil, Bolivia, and Argentina. In Africa, suitability losses could occur in South Africa, Zimbabwe, Angola, Zambia, Tanzania, Kenya, Uganda, Rwanda, Ethiopia, and Madagascar. Europe may also experience some reductions in climate suitability, particularly in Portugal and Spain. Additionally, Kazakhstan, Iran, Mongolia, Indonesia, Australia, and parts of China’s *B. napus*-producing regions might face reductions in climate suitability for *B. napus* (Fig. 3f). Notably, while some regions in China that currently produce *B. napus* may experience reduced suitability in the future, other regions within the country may see increased climatic suitability.

**Figure 4:**
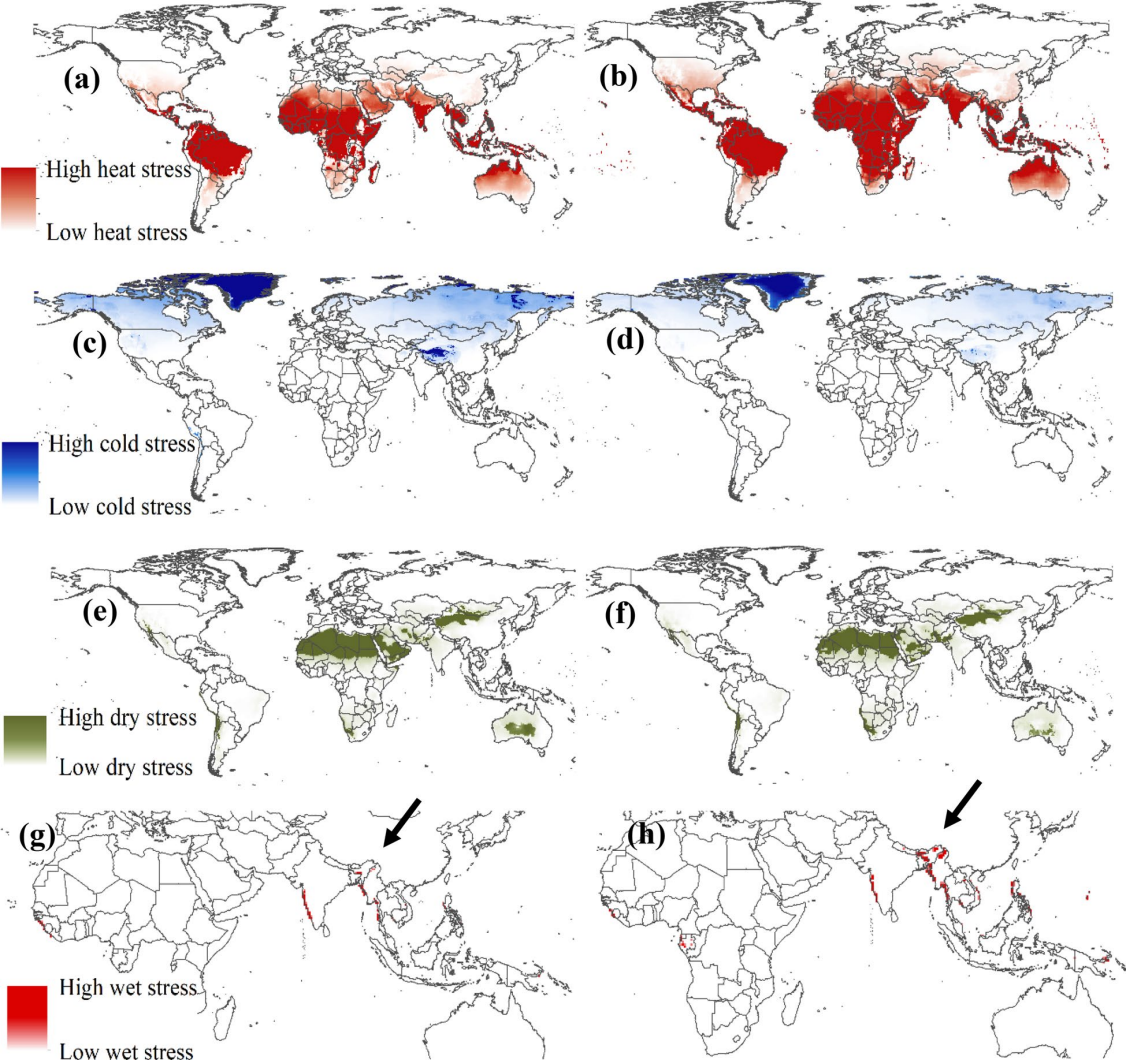
Projection of stress indices for *Brassica napus* in the world in different years (**a**) heat stress in the present time (**b**) heat stress in 2040–2059 under scenarios RCP 8.5 (**c**) cold stress in the present time (**d**) cold stress in 2040–2059 under scenarios RCP 8.5 (**e**) Dry stress in the present time (**f**) Dry stress in 2040–2059 under scenarios RCP 8.5; (**g**) wet stress in Africa and Asia present time (**h**) wet stress in Africa and Asia under in 2040–2059 scenarios RCP 8.5.

Figure 4 depicts changes in heat, cold, drought, and moisture stress for *B. napus* with climate change in the RCP 8.5 scenario between 2040 and 2059, based on the Access 1.0 and CNRM-CM5 models. Figure 4a,b demonstrate an increase in heat stress in certain South American and Central American countries, parts of the United States, various African countries, parts of China, and Australia. Figure 4c,d indicate a reduction in cold stress across a significant portion of Canada and Russia. Furthermore, parts of China currently experiencing cold stress for *B. napus* may see a notable decrease in cold stress in the future. Drought stress (Fig. 4e,f) and moisture stress (Fig. 4g,h) did not exhibit significant changes in the climate change scenario for *B. napus*. However, an increase in drought stress was observed in certain regions of China, while a reduction in moisture stress was noted in Australia (Fig. 4e,f). Increased moisture stress was observed only in specific territories in India and Bangladesh (Fig. 4g,h). These findings provide insights into the potential changes in climate suitability, as well as heat, cold, drought, and moisture stress for B.

## Discussion

The countries with the highest number of *B. napus* occurrence records (Fig. 1a) were Canada, the United States, and the European continent. These regions have extensive areas with suitable climates for *B. napus* cultivation (Fig. 1b) and are characterized by consistently low temperatures throughout the year. The average annual temperatures in these areas are 6.5 °C in Canada^58^, 11.1 °C in the United States^59^, and 10 °C in the European Union^60^. The favorable low temperatures in these regions promote the development of *B. napus*^48^. However, regions with warmer temperatures, such as most territories in Brazil, the African continent, and central Australia, exhibited a lack of occurrence records and predicted unsuitability (EI = 0) for *B. napus* cultivation. These findings can be attributed to the sensitivity of *B. napus* to high temperatures, especially during the post-anthesis period^61^. High temperatures can lead to reduced stem length and thickness, increased leaf thickness, and significant biomass reduction in *B. napus* compared to plants grown in milder temperatures^12^. Additionally, under high-temperature conditions, *B. napus* may exhibit abnormal stem elongation, increasing the risk of lodging due to rapid initial growth^14^.

Among the various environmental factors influencing *B. napus* development, air temperature stands out as the most critical variable for regulating its growth and development^48^. The sensitivity analysis conducted on the model's parameters in our study confirmed the temperature's relevance to *B. napus* climatic requirements. Regardless of the suitability category, parameters related to air temperatures were found to be the most sensitive. In the context of climate change, heat stress emerged as the primary factor contributing to the reduction in climate suitability for *B. napus* growth. Countries experiencing a decrease in climatic suitability for *B. napus* in the future also exhibited an increase in the heat stress index.

The rise in heat stress poses a significant constraint on *B. napus* cultivation in several major producing countries or regions, including China^12^. According to Hu et al.^62^, *B. napus* is the most important oilseed crop and the fourth largest crop in China, with a production of 14.7 million tons in 2014/2015. In China, *B. napus* is primarily cultivated in the Yangtze River basin, characterized by an average annual temperature ranging from 14 to 20 °C and annual rainfall between 1000 and 1400 mm^63^. Moreover, *B. napus* cultivation has rapidly expanded to arid and semi-arid regions in northwest China^64^, where the average annual temperature is 7.4 °C^65^ to 9.1 °C^66^, and annual rainfall ranges from 250 to 550 mm^65^. With climate change, these regions where *B. napus* is currently grown may undergo significant climate changes, leading to potential shifts in suitability for cultivating this species in the future^67^. Therefore, it is crucial to implement adaptation strategies in agricultural systems to mitigate the adverse effects of climate change on crops^68^.

In our model's predicted future scenario of climate change, the reduction in cold stress is associated with the expansion of climatically suitable areas for *B. napus* development. Currently, certain regions in Canada, Russia, and China experience high levels of cold stress, rendering them unsuitable for *B. napus* cultivation. However, in the future (2040–2059) under the RCP 8.5 scenario, cold stress is projected to decrease significantly in these regions, leading to increased climatic suitability for *B. napus*. As temperatures rise due to climate change, areas with initially low temperatures will become warmer, creating more favorable conditions for agricultural production. The impacts of climate change on the agroecosystem will vary across regions, with some experiencing positive effects and others negative^69^.

The effect of future increases in atmospheric carbon dioxide (CO_2_) on plant development has been a subject of numerous studies investigating the impacts of climate change on agricultural production^12,69,70^. In our study, the observed changes in climatic suitability for *B. napus* in different regions around the world could also be influenced by the projected increase in atmospheric CO_2_. These findings suggest that further research on the developmental response of *B. napus* to CO_2_ concentrations would be valuable. According to Van Vuuren et al.^56^, the average CO_2_ concentration for the RCP 8.5 scenario during the period 2040–2059 is projected to reach 540 ppm. Qaderi et al.^12^ observed that elevated atmospheric CO_2_ concentrations mitigate the impacts of water and heat stress on *B. napus* plants. Higher CO_2_ levels result in reduced stomatal opening in some plants, leading to decreased leaf transpiration and increased photosynthesis and water use efficiency. Consequently, with increased biomass production and reduced evapotranspiration, the growth and yield of most agricultural plants are expected to increase^69^.

## Future recommendations

*Brassica napus* holds the potential to serve as an alternative crop in regions currently facing unsuitable conditions for its growth due to various stresses. To achieve this, ongoing research efforts are focused on variety improvement, such as the *B. napus* tropicalization project. The aim of tropicalization is to develop *B. napus* varieties that are better adapted to tropical conditions, exhibiting enhanced heat tolerance, reduced sensitivity to photoperiod, and decreased cold hour requirements for flowering. Although research in this area is still underway, promising results have been obtained in experimental fields^71^. Given the potential consequences of climate change on *B. napus*, it is crucial to conduct more frequent studies on its improvement. By doing so, we can enhance the resilience of *B. napus* to changing climatic conditions, as demonstrated in this research.

## Research limitations

In this study, the CLIMEX modeling approach focused solely on the climatic conditions required for the development of *Brassica napus*. It did not take into account factors such as pests, natural enemies, interspecific competition, edaphic conditions (e.g., soil type), and genetic improvements in the species. Therefore, it is important to complement modeling studies with field studies to enhance the accuracy and applicability of the results. The findings of our study are particularly valuable for guiding management practices, identifying areas prone to drought where irrigation will be necessary, determining changes in cultivation areas, and facilitating the development of locally adapted *B. napus* varieties. While CLIMEX serves as a useful tool for climate niche modeling, it does have certain limitations that users should consider: (1) Simplified ecological assumptions: CLIMEX models rely on ecological assumptions that may not always accurately represent real-world conditions. For instance, the models assume species are in equilibrium with their environments and can only exist within a narrow range of climate conditions. These assumptions may not hold true in all cases, leading to potential inaccuracies in predictions. (2) Limited data input: CLIMEX models require both climate data and species occurrence data to generate predictions. However, data availability can be limited for certain regions or species, which may result in incomplete or less accurate models. (3) Lack of spatial dynamics: CLIMEX models are static and do not consider changes in species distributions over time. Consequently, these models may not fully capture how species distributions will shift in response to climate change. (4) Uncertainty in future climate projections: CLIMEX models rely on climate data to generate predictions, and future climate projections inherently carry uncertainties. The accuracy of the models can be influenced by the reliability and precision of the climate data used. (5) Limited scope: CLIMEX is primarily designed for modeling the potential geographic range of plant and animal species. It may not be well-suited for modeling other ecological aspects, such as species interactions or ecosystem processes. Considering these limitations, it is essential to interpret CLIMEX modeling results in conjunction with other sources of information and approaches to gain a more comprehensive understanding of the potential impacts of climate change on *Brassica napus* and its ecological dynamics.

## Conclusion

*Brassica napus*, despite its favorable climatic requirements, is currently not cultivated in several countries and territories that possess suitable conditions for its growth. Consequently, it holds great promise as a crop for oil extraction in these regions. However, the increasing temperatures associated with climate change can pose significant challenges to *B. napus* production in various countries and regions worldwide. Notably, areas that currently lack climatic suitability for the crop may become suitable as temperatures rise. This could have a substantial impact on *B. napus* cultivation due to its high sensitivity to thermal parameters.

In summary, climate change can exert both positive and negative influences on *Brassica napus* production, contingent upon the specific conditions prevailing in the cultivation region. Some potential negative impacts of climate change on *B. napus* include: (a) Alterations in temperature and rainfall patterns that can disrupt the timing and success of seed germination and crop growth. Drought conditions can result in reduced seed yield and compromised seed quality. (b) Extreme weather events like floods, hurricanes, and heat waves, which can cause damage or destruction to crops. (c) Increased frequency and severity of pests and diseases, leading to crop damage and decreased yields. On the other hand, certain potential positive impacts of climate change on *B. napus* include: (d) Higher temperatures and elevated carbon dioxide concentrations, which can facilitate accelerated growth and increased yield. (e) Longer growing seasons that may enable multiple harvests. (f) Expansion of the crop range due to changes in climatic conditions. Overall, the implications of climate change on *B. napus* production will be influenced by numerous factors, such as the specific region of cultivation, the severity and frequency of extreme weather events, and the ability of farmers to adapt to changing conditions through effective crop management practices.

### Supplementary Information

Supplementary Information.

## Data Availability

The authors confirm that the data supporting the findings of this study are available within the article and its supplementary materials.
