# Supplementary material for: Forecasting Brassica napus production under climate change with a mechanistic species distribution model
Source: Sci Rep. 2023 Aug 4;13:12656. doi: 10.1038/s41598-023-38910-3 (PMC10403512; doi:10.1038/s41598-023-38910-3)
Supplement: Supplementary file 1 — Supplementary Information. [file 41598_2023_38910_MOESM1_ESM.docx]

*Brassica napus* distribution

*Brassica napus* distribution from literature^1-28^

References

1 Luz, G. L. d. *et al.* Temperatura base inferior e ciclo de híbridos de canola. *Ciência Rural.* **42**, 1549-1555; https://doi.org/10.1590/S0103-84782012000900006 (2012).

2 Garcia, L. C. *et al.* Custo e lucro da produção de canola e milho nos Estados Unidos da América e Brasil. *Revista Conexão UEPG.* **17**, 1-17; https://doi.org/10.5212/Rev.Conexao.v.17.16805.013 (2021).

3 Rosa, A. d. S., Blochtein, B., Ferreira, N. & Witter, S. *Apis mellifera* (Hymenoptera: Apidae) as a potential *Brassica napus* pollinator (cv. Hyola 432)(Brassicaceae), in Southern Brazil. *Brazilian Journal of Biology.* **70**, 1075-1081; https://doi.org/10.1590/S1519-69842010000500024 (2010).

4 Chambo, E. D. *et al.* Pollination of rapeseed (*Brassica napus*) by Africanized honeybees (Hymenoptera: Apidae) on two sowing dates. *Anais da Academia Brasileira de Ciências.* **86**, 2087-2100; https://doi.org/10.1590/0001-3765201420140134 (2014).

5 Santos, R. F., Borsoi, A., Secco, D., de Souza, S. N. M. & Frigo, E. P. Nitrogen and sulfur sources in the culture of *Brassica napus* L. var. oleifera. *Journal of Food, Agriculture & Environment.* **10**, 516-518; https://www.researchgate.net/publication/268393580 (2012).

6 Mussury, R. M. & Fernandes, W. D. Studies of the floral biology and reproductive system of *Brassica napus* L.(Cruciferae). *Brazilian Archives of Biology and Technology.* **43**, 111-117; https://doi.org/10.1590/S1516-89132000000100014 (2000).

7 Fuzaro, L. *et al.* Floral visitors of canola (*Brassica napus* L.) hybrids in Cerrado Mineiro region, Brazil. *Arquivos do Instituto Biológico.* **86**; https://doi.org/10.1590/1808-1657001312018 (2019).

8 Arrúa, M. M. *et al.* Agronomic characteristics and oil content of different genotypes of canola (*Brassica napus* L. var. oleifera). *African Journal of Agricultural Research.* **12**, 2002-2007; https://doi.org/10.5897/AJAR2016.12023 (2017).

9 Gregoire, P., Rosset, J. D. & Gulden, R. H. Volunteer *Brassica napus* (L.) interference with soybean [*Glycine max* (L.) Merr.]: management thresholds, plant growth, and seed return. *Canadian Journal of Plant Science.* **101**, 556-567; https://doi.org/10.1139/cjps-2020-0258 (2021).

10 Gu, X.-B., Li, Y.-N. & Du, Y.-D. Effects of ridge-furrow film mulching and nitrogen fertilization on growth, seed yield and water productivity of winter oilseed rape (*Brassica napus* L.) in Northwestern China. *Agricultural Water Management.* **200**, 60-70; <https://doi.org/10.1016/j.agwat.2018.01.001> (2018).

11 Ilyas, M. *et al.* Genetic divergence in *Brassica napus* L. germplasm as determined by quantitative attributes. *Pakistan Journal of Botany.* **50**, 1039-1045; https://iris.uniroma1.it/handle/11573/1637695 (2018).

12 Kang, L., Li, P., Wang, A., Ge, X. & Li, Z. A novel cytoplasmic male sterility in *Brassica napus* (inap CMS) with carpelloid stamens via protoplast fusion with Chinese woad. *Frontiers in Plant Science.* **8**, 529; https://doi.org/10.3389/fpls.2017.00529 (2017).

13 Li, Y. *et al.* Accumulation, interaction and fractionation of fluoride and cadmium in sierozem and oilseed rape (*Brassica napus* L.) in northwest China. *Plant Physiology and Biochemistry.* **127**, 457-468; https://doi.org/10.1016/j.plaphy.2018.04.017 (2018).

14 Liu, Q. *et al.* Yield loss of oilseed rape (*Brassica napus* L.) under nitrogen deficiency is associated with under-regulation of plant population density. *European Journal of Agronomy.* **103**, 80-89; https://doi.org/10.1016/j.eja.2018.11.006 (2019).

15 Morales Moreira, Z. P., Helgason, B. L. & Germida, J. J. Environment has a stronger effect than host plant genotype in shaping spring *Brassica napus* seed microbiomes. *Phytobiomes Journal.* **5**, 220-230; https://doi.org/10.1094/PBIOMES-08-20-0059-R (2021).

16 Page, E. R., Meloche, S., Moran, M., Caldbeck, B. & Barthet, V. Effect of seeding date on winter canola (*Brassica napus* L.) yield and oil quality in southern Ontario. *Canadian Journal of Plant Science.* **101**, 490-499; https://doi.org/10.1139/cjps-2020-0220 (2021).

17 Qi, W. *et al.* Physiological and biochemical mechanisms and cytology of cold tolerance in *Brassica napus*. *Frontiers in Plant Science.* **11**, 1241; https://doi.org/10.3389/fpls.2020.01241 (2020).

18 Soltani, E., Gonzalez-Andujar, J. L., Oveisi, M. & Salehi, N. Development and validation of a predictive model for seedling emergence of volunteer canola (*Brassica napus*) under semi-arid climate. *International Journal of Plant Production.* **12**, 53-60; https://doi.org/10.1007/s42106-017-0006-7 (2018).

19 Soroka, J., Weiss, R., Grenkow, L. & Olfert, O. Relationships among root maggot (Delia spp., Diptera: Anthomyiidae) infestation, root injury, and seed yields of canola *Brassica napus* L. and *Brassica rapa* L. *Canadian Journal of Plant Science.* **100**, 575-591; https://doi.org/10.1139/cjps-2019-0278 (2020).

20 Taye, Z. M. *et al.* Core and differentially abundant bacterial taxa in the rhizosphere of field grown *Brassica napus* genotypes: implications for canola breeding. *Frontiers in Microbiology.* **10**, 3007; https://doi.org/10.3389/fmicb.2019.03007 (2020).

21 Tian, C. *et al.* Balanced fertilization under different plant densities for winter oilseed rape (*Brassica napus* L.) grown on paddy soils in Southern China. *Industrial crops and products.* **151**, 112413; https://doi.org/10.1016/j.indcrop.2020.112413 (2020).

22 Udall, J. A., Quijada, P. A., Polewicz, H., Vogelzang, R. & Osborn, T. C. Phenotypic effects of introgressing Chinese winter and resynthesized *Brassica napus* L. germplasm into hybrid spring canola. *Crop science.* **44**, 1990-1996; https://doi.org/10.2135/cropsci2004.1990 (2004).

23 Wang, H. *et al.* QTL and candidate gene identification for silique length based on high-dense genetic map in *Brassica napus* L. *Frontiers in plant science.* **10**, 1579; https://doi.org/10.3389/fpls.2019.01579 (2019).

24 Xue, L. *et al.* Botrytis pseudocinerea, a new pathogen causing gray mold on *Brassica napus* in China. *Plant Disease.* **103**, 367-367; https://doi.org/10.1094/PDIS-04-18-0688-PDN (2019).

25 Yu, R., Ji, J., Yuan, X., Song, Y. & Wang, C. Accumulation and translocation of heavy metals in the canola (*Brassica napus* L.)—soil system in Yangtze River Delta, China. *Plant and soil.* **353**, 33-45; https://doi.org/10.1007/s11104-011-1006-5 (2012).

26 Zhang, C.-J. *et al.* Assessment of potential environmental risks of transgene flow in smallholder farming systems in Asia: *Brassica napus* as a case study in Korea. *Science of the Total Environment.* **640**, 688-695; https://doi.org/10.1016/j.scitotenv.2018.05.335 (2018).

27 Zhang, H., Berger, J. D. & Milroy, S. P. Genotype× environment interaction studies highlight the role of phenology in specific adaptation of canola (*Brassica napus*) to contrasting Mediterranean climates. *Field Crops Research.* **144**, 77-88; https://doi.org/10.3389/fpls.2021.686332 (2013).

28 Gu, X. *et al.* Ridge-furrow full film mulching: an adaptive management strategy to reduce irrigation of dryland winter rapeseed (*Brassica napus* L.) in northwest China. *Agricultural and Forest Meteorology.* **266**, 119-128; https://doi.org/10.1016/j.agrformet.2018.12.009 (2019).
